# Prognostic value of albumin‐related ratios in HBV‐associated decompensated cirrhosis

**DOI:** 10.1002/jcla.24338

**Published:** 2022-03-17

**Authors:** Tan Zhang, Bin Ye, JianJiang Shen

**Affiliations:** ^1^ Department of Clinical Laboratory Shengzhou People's Hospital Shengzhou Branch of the First Affiliated Hospital of Zhejiang University Shengzhou China; ^2^ Department of Critical Care Medicine Shengzhou People's Hospital Shengzhou Branch of the First Affiliated Hospital of Zhejiang University Shengzhou China

**Keywords:** albumin‐related *ratios*, decompensated cirrhosis, hepatitis B virus, prognosis

## Abstract

**Background:**

Identification of effective and accurate prognostic biomarkers for hepatitis B virus‐associated decompensated cirrhosis (HBV‐DeCi) is challenging. This study was designed to determine and compare the prognostic value of albumin‐related *ratios* (blood urea nitrogen‐to‐albumin ratio [BAR], C‐reactive protein‐to‐albumin ratio [CAR], prothrombin time‐international normalized ratio‐to‐albumin ratio [PTAR], neutrophil count‐to‐albumin ratio [NAR], and D‐dimer‐to‐albumin ratio [DAR]) in HBV‐DeCi patients.

**Methods:**

We retrospectively recruited 161 HBV‐DeCi patients. Receiver operating characteristic curve, DeLong test, and Cox regression analyses were used to estimate and compare the predictive value of these five albumin‐related *ratios* and Model for End‐Stage Liver Disease (MELD) score.

**Results:**

A total of 29 (18.0%) patients had died 30 days after admission. The prognostic roles of CAR, DAR, PTAR, NAR, and BAR in HBV‐DeCi were different. CAR, PTAR, NAR, and BAR were significantly higher in non‐survivors compared with survivors. However, DAR did not differ between the two groups. The predictive power of BAR was superior to that of the other four albumin‐related biomarkers and similar to that of MELD score. On multivariate analysis, BAR and MELD score were identified as independent prognostic factors, and the combination of BAR and MELD score may improve the prognostic accuracy in HBV‐DeCi.

**Conclusion:**

The present findings suggest that BAR may be a simple and useful prognostic tool to predict mortality in HBV‐DeCi patients.

## Graphical abstract

ROC curve analyses for albumin‐related biomarkers and MELD score for prediction of s30‐day mortality in HBV‐DeCi patients

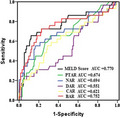

## 1 INTRODUCTION

In Asia, hepatitis B virus (HBV) is the major cause of liver cirrhosis.^1^ Decompensated cirrhosis (DeCi) is a terminal liver disease characterized by various complications and associated with dramatically reduced survival.^2^, ^3^, ^4^ Liver transplantation is the most effective therapy for HBV‐DeCi. However, the lack of available donors, potential for immunological rejection, and substantial cost restricts its application.^5^ Therefore, effective clinical risk assessment and stratification of HBV‐DeCi patients would be useful to achieve timely management and decrease mortality.

The Model for End‐Stage Liver Disease (MELD) score is the most commonly used scoring system for liver function assessment and serves as an independent risk predictor for prognosis. However, the score requires complex calculations and is inconvenient for routine practice. Furthermore, the score is affected by diuretics, bleeding, and ascites, and thus its prediction accuracy is limited and unsatisfactory.^6^ In clinical practice, the prognosis of HBV‐DeCi patients is difficult to predict because it is affected by many unstable factors, including inflammation, complications, and therapeutic options. These factors may be associated with the complex pathophysiological mechanism of HBV‐DeCi. Consequently, it is necessary to combine relevant parameters and construct models that can effectively predict the prognosis of HBV‐DeCi patients.

Serum albumin is considered a biomarker of malnutrition–inflammation syndrome, and there is emerging data that hypoalbuminemia is related to worse survival in intensive care unit patients.^7^, ^8^, ^9^ Albumin has multiple functions and plays important roles in liver disease severity, progression, and prognosis. First, albumin is a negative acute‐phase reactant and decreases in response to inflammation, thereby serving as an inflammation marker. Second, albumin is synthesized in the liver and can reflect the liver synthetic function, an important indicator for assessment of hepatic function. Third, albumin is a marker that reflects nutritional status, and low albumin is often associated with malnutrition. Decreased albumin is a common complication in cirrhotic patients that can lead to ascites and hepatic encephalopathy, and even a tendency toward spontaneous bacterial peritonitis, and has a negative impact on prognosis.^10^, ^11^ Recently, the clinical usefulness of albumin‐related biomarkers available from routine testing has been reported in various diseases, including liver diseases, such as C‐reactive protein‐to‐albumin ratio (CAR),^12^, ^13^, ^14^ D‐dimer‐to‐albumin ratio (DAR),^15^ prothrombin time‐international normalized ratio‐to‐albumin ratio (PTAR),^16^, ^17^, ^18^ neutrophil count‐to‐albumin (NAR),^19^, ^20^, ^21^ and blood urea nitrogen‐to‐albumin ratio (BAR).^22^, ^23^, ^24^, ^25^ However, no studies have investigated the differences in prognostic value of these albumin‐related *ratios* in HBV‐DeCi patients. Therefore, we conducted this retrospective study to determine and compare the prognostic value of these albumin‐related *ratios* and their clinical utility in HBV‐DeCi patients in China.

## 2 MATERIALS AND METHODS

### 2.1 Study population participants

We recruited HBV‐DeCi patients between March 30, 2019, and May 30, 2021. This study design was approved by the Ethics Committee of the Shengzhou People's Hospital. DeCi was defined by clinical, biochemical, and endoscopic findings, together with presence of ascites, gastrointestinal bleeding, hepatorenal syndrome, or hepatic encephalopathy.^3^ All patients presented with clinical manifestations of decompensated liver disease for the first time. The inclusion criteria were (1) HBsAg positivity for >6 months and (2) age between 18 and 75 years. The exclusion criteria were (1) co‐infection with HIV; (2) malignant tumors; (3) underlying liver disease (e.g., other viral hepatitis and alcohol‐ or drug‐related liver diseases); and (4) complication with cardiac diseases or blood‐system diseases. All patients received antiviral therapy from the start date. The primary outcome was 30‐day survival.

### 2.2 Clinical data collection

Demographic and clinical parameters, including gender, age, laboratory variables (total bilirubin, total protein, albumin, blood urea nitrogen [BUN], creatinine, aspartate aminotransferase, alanine aminotransferase, C‐reactive protein [CRP], white blood count [WBC], platelet count, hemoglobin, neutrophil count, plasma D‐dimer, and international normalized ratio [INR]), and HBV‐DNA levels, as well as hepatitis B e antigen status, were recorded at admission. Severity of liver disease and prognosis were assessed using the MELD score.^26^

### 2.3 Calculation of the formulas

PTAR = PT‐INR/albumin (g/dl)
NAR = Neutrophil count (× 10⁹/L)/albumin (g/dL)
CAR = CRP (mg/L)/albumin (g/dl)
DAR = D‐dimer (mg/L FEU)/albumin (g/dl)
BAR = BUN (mmol/L)/albumin (g/dl)
MELD score = 3.78 × ln (total bilirubin, mg/dl) + 11.2 × ln (INR) + 9.57 × ln (creatinine, mg/dl) + 6.4

### 2.4 Statistical analysis

Statistical analyses were performed using SPSS version 19.0 and MedCalc version 15.2.1. Statistical significance was defined at *p* < 0.05. Baseline and clinical characteristics were presented as median with interquartile range (IQR) or number. Differences in variables between non‐survivors and survivors at 30 days were analyzed by the Mann–Whitney U test or the chi‐square test. Correlations between MELD score and CAR, DAR, PTAR, NAR, and BAR were analyzed by Spearman's analysis. Logistic regression models were performed to explore the associations of MELD score, CAR, DAR, PTAR, NAR, and BAR with poor outcomes. The predictive accuracy of each factor was determined by receiver operating characteristic (ROC) curve analysis using the area under the ROC curve (AUC). Comparisons between AUCs were performed using the De‐Long test. The sensitivity, specificity, positive predictive value (PPV), and negative predictive value (NPV) were compared among the factors.

## 3 RESULTS

### 3.1 Baseline characteristics

**FIGURE 1:**
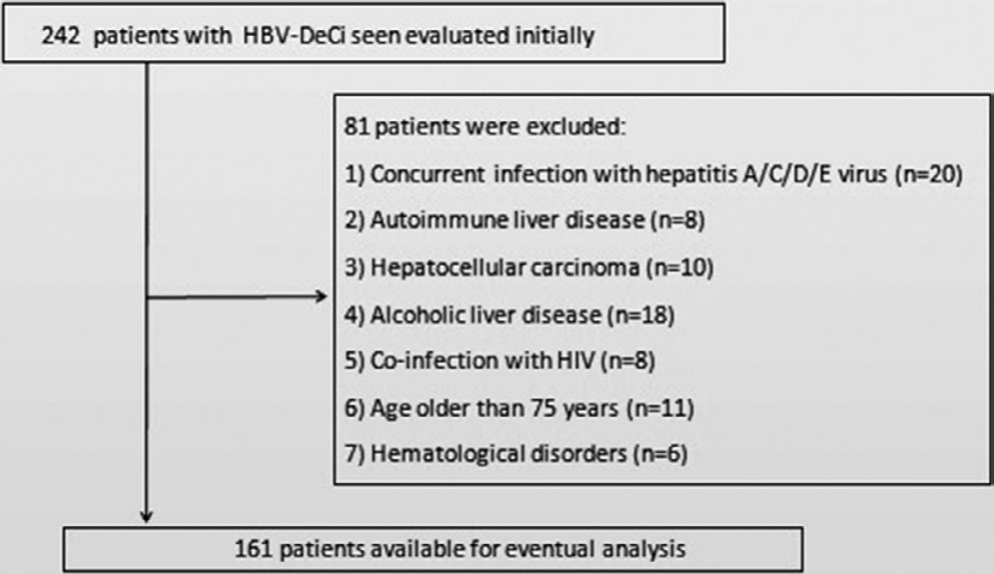
Flow diagram of the patients enrolled in this study and the reasons for exclusion

**FIGURE 2:**
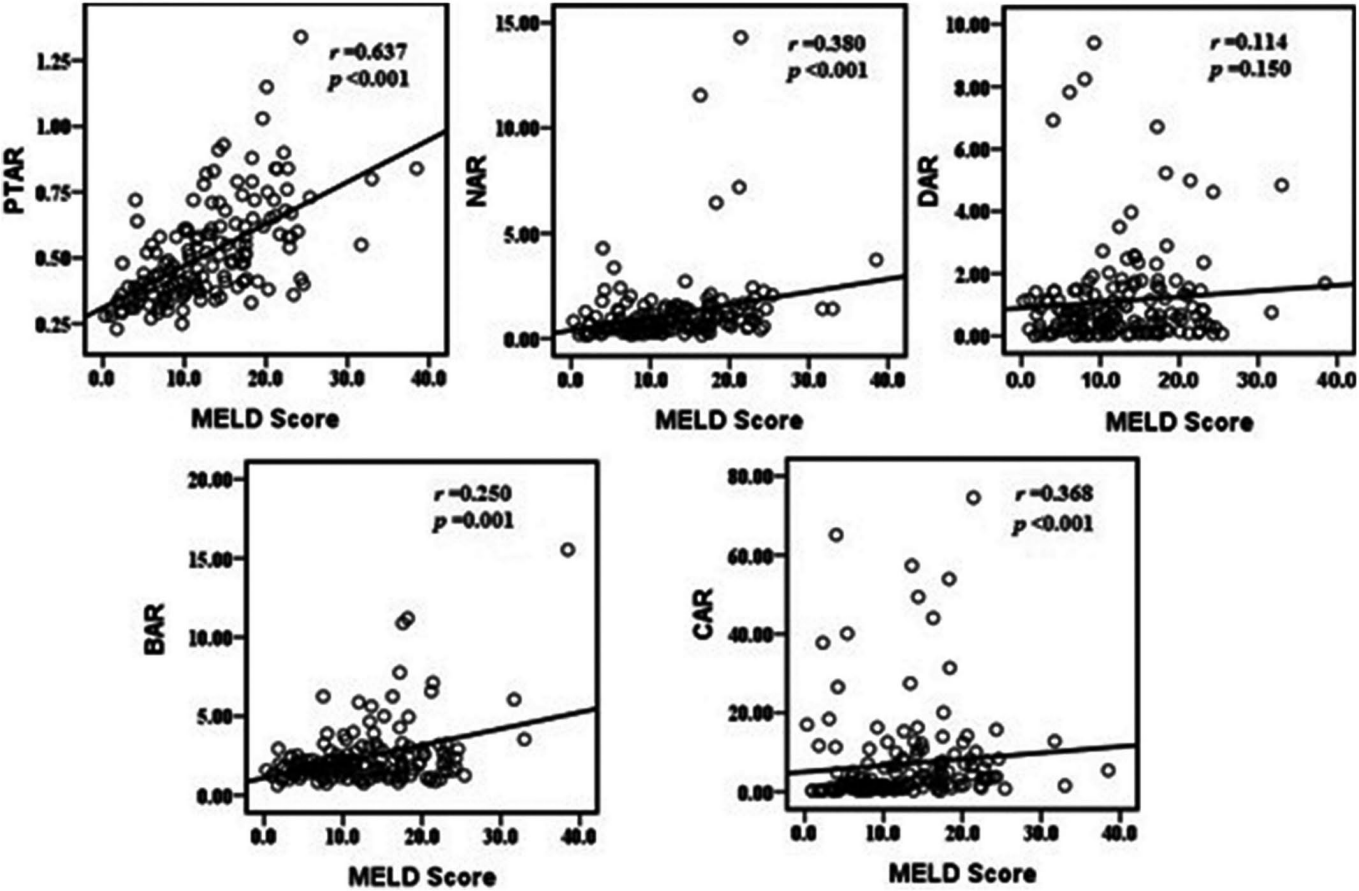
Correlations of MELD score with PTAR (*r* = 0.637, *p* < 0.001), NAR (*r* = 0.380, *p* < 0.001), DAR (*r* = 0.114, *p* = 0.150), CAR (*r* = 0.368, *p* < 0.001), and BAR (*r* = 0.250, *p* = 0.001) in HBV‐DeCi patients

A total of 161 HBV‐DeCi patients were enrolled in the study after screening of 242 patients (Figure 1). The main causes of admission were ascites (*n* = 120, 74.5%), gastrointestinal bleeding (*n* = 36, 22.5%), hepatorenal syndrome (*n* = 22, 14.0%), and encephalopathy (*n* = 10, 6.2%). Twenty‐four patients (15.0%) had more than one feature of decompensation at presentation. The median values of CAR, DAR, PTAR, NAR, and BAR at enrollment were 2.48 (IQR, 1.04–7.48), 0.69 (IQR, 0.23–1.42), 0.47 (IQR, 0.38–0.61), 0.72 (IQR, 0.49–1.31), and 1.84 (IQR, 1.39–2.58), respectively. Positive correlations were found between MELD score and PTAR, NAR, CAR, and BAR (all *p* < 0.05), while DAR was not correlated with MELD score (*p* = 0.150) (Figure 2).

In the study population, the 30‐day mortality rate was 18.0% (29/161). The cause of death was hepatic failure (*n* = 8), gastrointestinal bleeding (*n* = 8), encephalopathy (*n* = 7), hepatorenal syndrome (*n* = 5), and unknown (*n* = 1). The patients were divided into non‐survivors (*n* = 29) and survivors (*n* = 132). Table 1 shows the baseline characteristics in the two groups. Significant differences in total bilirubin, creatinine, INR, total protein, hemoglobin, CAR, PTAR, NAR, BAR, and MELD score were found between the two groups (all *p* < 0.05). Compared with survivors, non‐survivors were more likely to have ascites, hepatorenal syndrome, or encephalopathy (all *p* < 0.05).

**TABLE 1:** Comparisons of baseline characteristics between the survivors and non‐survivors

	All patients (*n* = 161)	Nonsurviving patients (*n* = 29)	Surviving patients (*n* = 132)	*p*
Gender (female/male)	34/127	8/21	26/106	0.489
Age (years)	53.0 (46.0–63.0)	55.0 (49.0–63.5)	52.0 (46.0–62.5)	0.337
Total protein (g/dl)	6.08 (5.60–66.7)	56.4 (50.8–64.2)	61.4 (56.7–66.9)	0.013
Albumin (g/dl)	3.06 (2.64–3.36)	3.00 (2.58–3.26)	3.09 (2.64–3.42)	0.300
Alanine aminotransferase (U/L)	30.0 (16.0–55.0)	38.0 (14.0–63.3)	30.0 (16.5–54.0)	0.741
Aspartate aminotransferase (U/L)	46.0 (28.0–74.0)	51.5 (30.5–113.5)	46.0 (28.0–72.8)	0.563
Serum creatinine (mmol/L)	72.0 (59.0–86.3)	92.0 (66.0–125.5)	69.0 (58.5–82.0)	0.002
Total bilirubin (μmol/L)	42.0 (19.0–118.0)	78.0 (46.3–215.0)	36.0 (18.0–101.0)	0.011
INR	1.37 (1.22–1.64)	1.64 (1.30–1.93)	1.33 (1.18–1.57)	0.002
PTAR	0.47 (0.38–0.61)	0.58 (0.42–0.77)	0.45 (0.37–0.58)	0.003
NAR	0.72 (0.49–1.31)	1.41 (0.55–2.19)	0.68 (0.45–1.15)	0.001
DAR	0.69 (0.23–1.42)	0.64 (0.32–1.86)	0.70 (0.22–1.36)	0.391
CAR	2.48 (1.04–7.48)	6.02 (1.50–12.54)	2.18 (0.98–6.92)	0.041
BAR	1.84 (1.39–2.58)	2.95 (2.12–5.92)	1.71 (1.32–2.26)	<0.001
Platelet count (×10⁹/L)	72.0 (43.8–114.3)	71.0 (53.5–132.3)	73.0 (43.0–113.0)	0.751
Hemoglobin (g/L)	102.0 (84.8–120.3)	93.0 (69.8–108.8)	104.5 (86.0–121.0)	0.011
WBC (×10⁹/L)	4.30 (2.70–5.90)	5.10 (2.70–8.50)	4.00 (2.70–5.55)	0.014
CRP (mg/L)	7.70 (3.07–20.52)	16.20 (4.08–34.48)	6.30 (2.85–16.60)	0.033
Ascites (yes/no)	120/41	26/3	3/129	<0.001
Gastrointestinal bleeding (yes/no)	36/125	8/21	28/104	0.617
Hepatorenal syndrome (yes/no)	22/139	8/21	14/118	0.035
Encephalopathy (yes/no)	10/151	7/22	3/129	<0.001
HBV‐DNA (Log_10_ IU/ml)	4.1 (3.3–8.0)	3.7 (3.3–6.1)	5.2 (3.8–9.0)	0.546
HBeAg‐positive (yes/no)	64/97	12/17	52/80	0.991
MELD score	11.6 (7.3–17.3)	20.3 (11.9–22.6)	10.6 (6.8–15.1)	<0.001

Note

Data are expressed as *n* or median (interquartile range).

Abbreviations: BAR, blood urea nitrogen‐to‐albumin ratio; CAR, C‐reactive protein‐to‐albumin ratio; DAR, D‐dimer‐to‐albumin ratio; INR, international normalized ratio; MELD score, Model for End‐Stage Liver Disease score; NAR, neutrophil count‐to‐albumin ratio; PTAR, prothrombin time‐international normalized ratio‐to‐albumin ratio; WBC, white blood count.

### 3.2 Utility of albumin‐related biomarkers for predicting mortality in HBV‐DeCi patients

**FIGURE 3:**
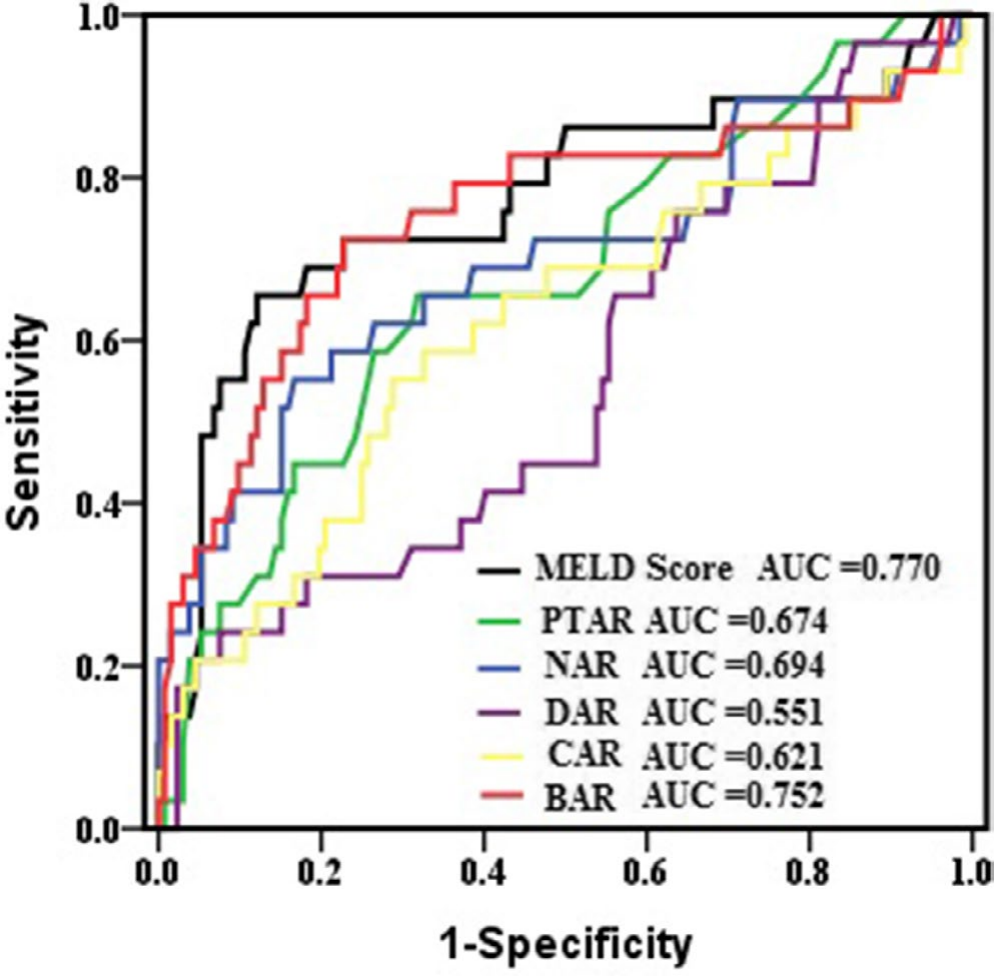
ROC curve analyses for albumin‐related ratios and MELD score for prediction of 30‐day mortality in HBV‐DeCi patients

Table 2 lists the relationships between the albumin‐related *ratios*, MELD score, WBC, CRP, and mortality. In univariate analyses, high MELD score, high CAR, high PTAR, high NAR, high BAR, high WBC, and high CRP were associated with mortality. In multivariate analyses, only high BAR and high MELD score were identified as independent factors for worse survival. Next, ROC curves were used to assess the discrimination ability of the factors for mortality (Figure 3). The AUCs for CAR and DAR for the prediction of mortality were not significant (both *p* > 0.05). The AUC for BAR was 0.752, which was higher than that for PTAR (0.674), NAR (0.694), DAR (0.551), and CAR (0.621), and comparable to that for MELD score (0.770) (Table 3). Among the six parameters, MELD score, NAR, and BAR had higher prognostic specificity than CAR and PTAR (87.9%, 83.3%, and 77.3% vs. 71.2% and 68.2%, respectively), while BAR had the highest prognostic sensitivity compared with MELD score, CAR, DAR, PTAR, and NAR (72.4% vs. 65.5%, 55.1%, 24.1%, 65.5%, and 55.2%, respectively). The highest PPV was observed for MELD score (54.3%), indicating that it was the best predictive factor for mortality, while MELD score and BAR had the best NPV (92.1% and 92.7%, respectively), indicating that they could be used to exclude mortality (Table 3). When BAR and MELD score were combined, the sensitivity and specificity became 75.8% and 84.1%, respectively, and the AUC increased to 0.815.

**TABLE 2:** Univariate and multivariate analyses of risk factors associated with mortality in HBV‐DeCi patients

	Univariable			Multivariable		
	Odds ratio	95% CI	*p*	Odds ratio	95% CI	*p*
MELD score	1.181	1.099–1.269	<0.001	1.160	1.072–1.257	<0.001
PTAR	19.709	2.565–151.438	0.004			
NAR	2.433	1.430–4.141	0.001			
DAR	1.211	0.975–1.504	0.084			
CAR	1.041	1.012–1.072	0.005			
BAR	1.677	1.287–2.185	<0.001	1.510	1.148–1.988	0.003
WBC (× 10⁹/L)	1.239	1.080–1.421	0.002			
CRP (mg/L)	1.018	1.005–1.030	0.005			

Abbreviations: BAR, blood urea nitrogen‐to‐albumin ratio; CAR, C‐reactive protein‐to‐albumin ratio; CI, confidence interval; DAR, D‐dimer‐to‐albumin ratio; MELD, Model for End‐Stage Liver Disease; NAR, neutrophil count‐to‐albumin ratio; PTAR, prothrombin time‐international normalized ratio‐to‐albumin ratio; WBC, white blood count.

**TABLE 3:** Prognostic accuracies of different scoring systems for predicting 30‐day mortality

	AUC	*p*	Cut‐off value	Sensitivity	Specificity	NPV	PPV
MELD score	0.770^a^	<0.001	17.5	65.5	87.9	92.1	54.3
PTAR	0.674^b^	0.003	0.53	65.5	68.2	90.0	31.1
NAR	0.694^c^	0.003	1.37	55.2	83.3	89.4	42.1
DAR	0.551^d^	0.408	2.35	24.1	92.4	84.7	41.0
CAR	0.621^e^	0.054	5.08	55.1	71.2	87.8	29.6
BAR	0.752^f^	<0.001	2.32	74.2	77.3	92.7	41.2

Note

a versus b: *p* = 0.073; a versus c: *p* = 0.190; a versus d: *p* = 0.004; a versus e: *p* = 0.024; a versus f: *p* = 0.808; b versus c: *p* = 0.750; b versus d: *p* = 0.056; b versus e: *p* = 0.383; b versus f: *p* = 0.339; c versus d: *p* = 0.016; c versus e: *p* = 0.172; c versus f: *p* = 0.425; d versus e: *p* = 0.325; d versus f: *p* = 0.002; e versus f: *p* = 0.089.

Abbreviations: AUC, area under curve; BAR, blood urea nitrogen‐to‐albumin ratio; CAR, C‐reactive protein‐to‐albumin ratio; CI, confidence interval; DAR, D‐dimer‐to‐albumin ratio; MELD, Model for End‐Stage Liver Disease; NAR, neutrophil count‐to‐albumin ratio; NPV, negative predictive value; PPV, positive predictive value; PTAR, prothrombin time‐international normalized ratio‐to‐albumin ratio.

## 4 DISCUSSION

Patients with HBV‐DeCi have a very high risk of mortality, and determining the prognosis of HBV‐DeCi is challenging. Current research has mainly focused on the prognostic value of albumin‐related biomarkers in these patients. To our knowledge, this is the first study to date that determines and compares the five albumin‐related *ratios* for poor survival prediction in HBV‐DeCi patients. We found that the prognostic roles of CAR, DAR, PTAR, NAR, and BAR in HBV‐DeCi patients were different. The major findings of the study are summarized below.

First, there is considerable evidence that inflammation is common in advanced cirrhotic patients and associated with adverse outcomes.^27^, ^28^ Neutrophil count and CRP are universal inflammatory markers, and the prognostic role of values based on these two inflammatory factors in HBV‐associated liver disease has been explored, including NAR (ratio of neutrophil count to albumin)^19^ and CAR (ratio of CRP to albumin).^12^ For example, Han et al.^19^ proposed that high NAR was linked to poor survival in HBV‐DeCi patients, while Huang et al.^12^ found that CAR was related to the prognosis of HBV‐DeCi patients and was better than MELD score for predicting HBV‐DeCi mortality. However, NAR and CAR were not identified as predictors of poor outcomes in the present multivariate analyses. This may be due to differences in the risk factors included in the regression analyses in the three studies. Notably, the AUC for CAR to predict mortality was not significant compared with the reference line (*p* = 0.054) in the present study. This may arise because CRP is influenced by multiple factors such as body mass index, weight loss, and smoking,^29^ meaning that it cannot effectively reflect the inflammatory status of the liver, the disease severity, or the prognosis of patients. Another possible reason is the relatively small number of patients included in our study.

Second, coagulopathy and hemorrhage risk are commonly seen in severe liver cirrhosis patients, because of decreased production of blood pro‐ and anti‐coagulant proteins in the liver and/or portal hypertension.^30^, ^31^ Recently, PTAR was reported as a novel method for liver function assessment in hepatocellular carcinoma.^17^ In addition, Gao et al.^18^ demonstrated that PTAR could predict short‐term mortality in cirrhotic patients, while Cai et al.^16^ showed that high PTAR was associated with poor prognosis in HBV‐DeCi patients. However, the present study did not identify PTAR as a predictor of mortality in HBV‐DeCi patients in multivariate analyses. Meanwhile, recent studies showed that elevated plasma D‐dimer can predict poor prognosis in patients with liver diseases.^32^, ^33^, ^34^ Küçükceran et al.^15^ found that DAR, as the ratio of D‐dimer to albumin, can serve as a valuable predictor for mortality in COVID‐19 patients.^15^ However, the present results showed that DAR was similar between non‐survivors and survivors among HBV‐DeCi patients. Moreover, we did not find that DAR was associated with mortality in univariate analyses, and its predictive ability was inferior to that of the other four markers.

The third and most important finding is that we identified BAR and MELD score as independent predictive factors for mortality and demonstrated that these two factors had excellent predictive value for 30‐day mortality in HBV‐DeCi patients (both AUC > 0.700). BUN is a surrogate biomarker for increased severity of renal or systemic illness. According to recent studies, BUN was associated with unfavorable outcomes in various settings, including acute heart failure,^22^ critically ill patients,^23^ acute aortic dissection,^24^ and pancreatitis.^25^ Furthermore, Yu et al.^35^ identified BUN and age as predictive factors for poor prognosis in advanced liver diseases using machine learning. Kidney dysfunction is a common comorbid condition in liver disease, and several reports have indicated that kidney dysfunction is linked to mortality in cirrhotic patients.^36^ Among the 161 patients in the present cohort, 22 (14.0%) suffered from hepatorenal syndrome. In addition, gastrointestinal bleeding has a high incidence in cirrhotic patients and carries a high risk of death and decreased hemoglobin in these patients.^37^ In the present study, hemoglobin was significantly lower in non‐survivors compared with survivors. Given the breakdown and subsequent absorption of blood via the gastrointestinal tract, elevated BUN is often seen in HBV‐DeCi patients. Meanwhile, albumin is a biomarker for malnutrition–inflammation syndrome. The combination of BUN and albumin may provide an effective and simple prognostic tool to predict poor outcomes in HBV‐DeCi patients. The present results showed that BAR was markedly higher in non‐survivors compared with survivors and positively correlated with MELD score. These findings indicate that high BAR is predictive for disease severity and progression in HBV‐DeCi patients. We hypothesize that the high BAR in HBV‐DeCi patients may represent the cumulative effects of several factors, including renal dysfunction, gastrointestinal bleeding, malnutrition, and inflammation; it may also reflect the conditions of the patients more comprehensively, thus reflecting the risk of mortality to a certain extent. Moreover, the combination of BAR and MELD score can add to the predictive power for mortality in HBV‐DeCi patients.

## 5 CONCLUSIONS

In summary, we evaluated five albumin‐related *ratios* (CAR, DAR, PTAR, NAR, and BAR) for the prediction of mortality in HBV‐DeCi patients. Our study suggests that BAR can be a simple, effective, and useful prognostic tool to predict poor outcomes in HBV‐DeCi patients and that use of a combination of BAR and MELD score can improve the prognostic accuracy. However, because this was a retrospective study, further prospective clinical trials are warranted to validate the present results.

## CONFLICT OF INTEREST

None of the authors have any commercial or other association that might pose a conflict of interest.

## Data Availability

The data are available upon reasonable request.
